# Age and Growth of Endangered Smalltooth Sawfish (*Pristis pectinata*) Verified with LA-ICP-MS Analysis of Vertebrae

**DOI:** 10.1371/journal.pone.0047850

**Published:** 2012-10-17

**Authors:** Rachel M. Scharer, William F. Patterson III, John K. Carlson, Gregg R. Poulakis

**Affiliations:** 1 Department of Biology, University of West Florida, Pensacola, Florida, United States of America; 2 National Marine Fisheries Service, Southeast Fisheries Science Center, Panama City Laboratory, Panama City Beach, Florida, United States of America; 3 Florida Fish and Wildlife Conservation Commission, Fish and Wildlife Research Institute, Charlotte Harbor Field Laboratory, Port Charlotte, Florida, United States of America; University of Canterbury, New Zealand

## Abstract

Endangered smalltooth sawfish (*Pristis pectinata*) were opportunistically sampled in south Florida and aged by counting opaque bands in sectioned vertebrae (n = 15). Small sample size precluded traditional age verification, but fish collected in spring and summer had translucent vertebrae margins, while fish collected in winter had opaque margins. Trends in Sr:Ca measured across vertebrae with laser ablation-inductively coupled plasma-mass spectrometry corresponded well to annual salinity trends observed in sawfish estuarine nursery habitats in south Florida, thus serve as a chemical marker verifying annual formation of opaque bands. Based on that finding and assumptions about mean birth date and timing of opaque band formation, estimated age ranged from 0.4 y for a 0.60 m total length (TL) male to 14.0 y for a 4.35 m TL female. Von Bertalanffy growth parameters computed from size at age data were 4.48 m for L_∞_, 0.219 y^−1^for k, and −0.81 y for t_0_. Results of this study have important implications for sawfish conservation as well as for inferring habitat residency of euryhaline elasmobranchs via chemical analysis of vertebrae.

## Introduction

Assessment of the population viability or threat of extinction for endangered species requires information on population dynamics, including vital rates of growth and mortality. Age estimates are critical for estimating both of those parameters, as well as for computing population viability models [1], [2]. Thus, implementation of conservation actions and successful recovery of endangered populations requires precise and accurate age information such that informed decisions on recovery strategies can be made.

Sawfish (Family Pristidae) populations have been declining worldwide and currently are among the most endangered marine fishes. The International Union for Conservation of Nature (IUCN) lists all extant sawfish species as critically endangered [3]. In the United States, the smalltooth sawfish, *Pristis pectinata*, was commonly found at the turn of last century in the coastal zone from Texas to North Carolina and throughout the Gulf of Mexico [4]. However, the population declined by approximately 95% in the 20^th^ Century, primarily due to fisheries bycatch and habitat loss, and today individuals are only regularly encountered in south Florida [5]–[8]. Because of this large population decline and range reduction, the U.S. distinct population segment of smalltooth sawfish was listed as endangered under the Endangered Species Act (ESA) in 2003 following a formal status review by the US National Marine Fisheries Service [9]. Subsequently a recovery plan was produced by scientists and managers that outlined specific recommendations to promote conservation and recovery of the remaining population and critical habitats were designated for juveniles [10].

The ability of resource managers to develop recovery strategies for smalltooth sawfish is severely limited by a lack of relevant scientific data for this species [9], [10]. At the time of its listing under the ESA, little life history information was available on smalltooth sawfish, thus it was assumed they followed similar patterns of growth as congeners for which life history parameters had been estimated. Population viability analysis required under the ESA further amplifies the need for life history data specific to smalltooth sawfish. To that end, Simpfendorfer et al. [11] produced the first estimates of smalltooth sawfish growth via analysis of juvenile length frequency and tag-recapture data, but they indicated growth estimates were uncertain beyond the juvenile stage [i.e., fish >2.2 m stretched total length (TL)].

The most common technique for aging in elasmobranchs is counting opaque bands in vertebrae centra [12]. Slow winter growth results in tighter opaque bands, while faster growth results in wider translucent bands. If this alternating banding pattern repeats annually, then fish age can be estimated by counting opaque bands. While annual formation of opaque bands has been validated or verified for numerous elasmobranch species, there are some species in which opaque bands do not form annually (e.g., [13]–[15]). Therefore, age verification or validation is imperative for demonstrating that the number of opaque bands reflects fish age [16]–[18].

Verification techniques typically require large sample sizes to examine seasonal trends in opaque band formation, while validation techniques typically require hard parts to be chemically marked with animals either held in captivity or tagged and released for later recapture (reviewed in Cailliet et al. [18]). Recently, Hale et al. [19] reported that calcium (Ca) and phosphorus (P) peaks assayed in round stingray, *Urobatis halleri*, vertebrae with laser ablation-inductively coupled plasma-mass spectrometry (LA-ICP-MS) corresponded to opaque zones, which they inferred was verification of annual formation of opaque bands. Such a technique may be ideal for verifying periodicity of opaque zone formation in endangered fishes, such as the smalltooth sawfish, for which small sample sizes typically would preclude application of traditional verification techniques, and for which sacrificing chemically marked fish for age validation would not be possible.

The goal of the current study was to estimate age and growth parameters for smalltooth sawfish. Age was estimated by counting opaque bands in sections of vertebrae centra. Verification of the annual periodicity of opaque band formation was performed via LA-ICP-MS analysis of vertebrae. Lastly, a von Bertalanffy growth function (VBGF) was fit to size at age data to estimate growth.

## Methods

Vertebrae were collected from naturally deceased smalltooth sawfish necropsied in south Florida (Figure by National Marine Fisheries Service (NMFS), Mote Marine Laboratory (MML), University of Florida (UFL), or Florida Fish and Wildlife Research Institute (FWRI) personnel. Samples were either archived in ethanol or stored dry prior to being analyzed. Vertebrae centra were cleaned of any adhering tissue with bleach and then sectioned (0.5 mm width) with a low-speed Isomet® saw. Opaque bands in each section, including those on the margin, were counted independently by two readers (RMS and JKC) under transmitted light with a stereo microscope (magnification  = 10–63x) attached to an image analysis system. Opaque bands were distinguished from checks in the margins by only counting bands that occurred in the corpus calcareum (edge) on one side of the section and extended through the intermedialia (middle) and back through the corpus calcaerum on the other side of the section. Average percent error (APE) was computed between reader counts with the method of Beamish and Fournier [20].

**Figure 1:**
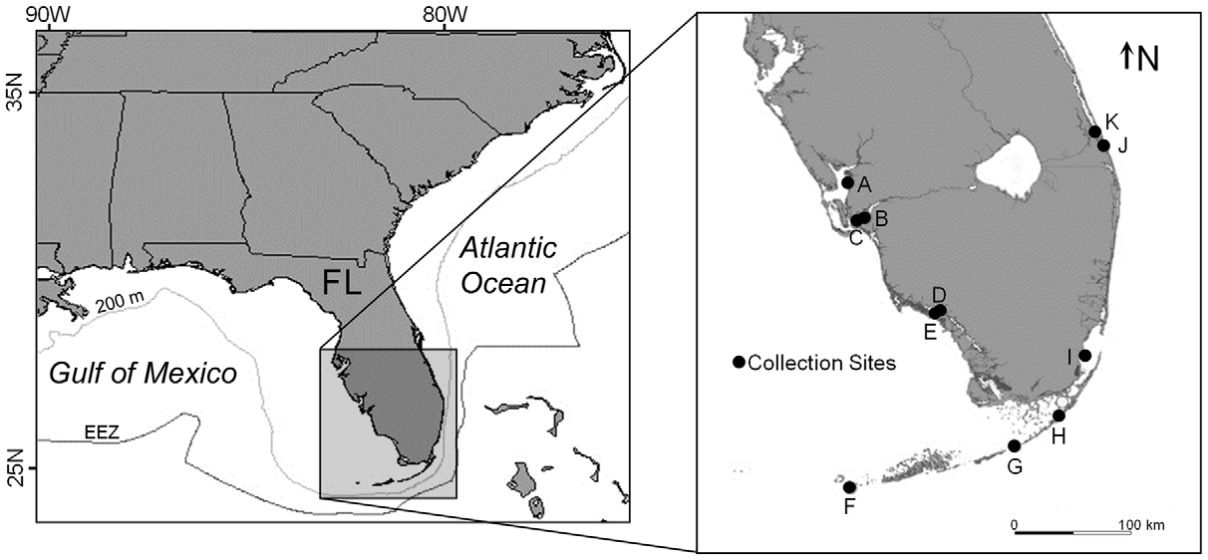
Map of locations in south Florida where smalltooth sawfish carcasses were opportunistically sampled. Location letters correspond to fish listed in Table 1.

### Age verification

An attempt was made to replicate the age verification method of Hale et al. [19] based on LA-ICP-MS analysis of sawfish vertebrae sections. Vertebrae that had been stored dry were sectioned and prepared for LA-ICP-MS analysis. Sections were placed in acid-leached polystyrene cell wells filled with 18.3 MΩ cm^−1^ ultrapure water. Cell wells were placed in a water bath in an ultrasonic cleaner for 1 h. Following ultrasonic cleaning, sections were rinsed with ultrapure water, placed in novel acid-leached cell wells, and then placed under a class-10 clean hood to air dry for 24 h. Once dry, sections were secured to microscope slides with double sided cellophane tape and placed in zipper seal plastic bags.

Vertebrae sections were analyzed for Ca, P, and strontium (Sr) with a New Wave Research UP 213 laser ablation system integrated with a Varian 820 quadrupole ICP-MS. The laser in this system is a solid state Nd:YAG laser with an output frequency of 213 nm and a maximum energy of 4 mJ. Each run consisted of a blank (1% ultrapure HNO_3_), pre-ablation and ablation of a standard, and then pre-ablation and ablation of six samples. The standard used was the United States Geological Survey's (USGS) MACS-3 solid calcium carbonate standard, which has certified concentration values for ^44^Ca and ^88^Sr but not for P. Pre-ablation scan speed was 30 µm sec^−1^, with a repetition rate of 10 hz and a spot size of 55 µm. Ablation scan speed was 10 µm sec^−1^, with a repetition rate of 10 hz and a spot size of 30 µm. Vertebra material vaporized by the laser was swept by He gas into the ICP-MS plasma. Element-specific count data from the ICP-MS detector were exported into an Excel® spreadsheet. Standard curves could not be used to convert count data to element concentrations because the MACS-3 standard is a solid carbonate standard with a single concentration per element. Ca and Sr concentrations were estimated from isotope-specific counts (^44^Ca and ^88^Sr) while correcting for blanks. Instrument drift was not an issue because the MACS-3 standard was analyzed prior to each sample. Sr:Ca data are presented as molar ratios, while count data alone are presented for P.

Detector count data for Ca and P were plotted versus vertebrae transect distance to replicate the age verification method of Hale et al. [19]. The degree to which Ca or P peaks corresponded to opaque bands was evaluated by overlaying the position of opaque bands on plots of Ca and P count data. A second verification method was performed in which trends in Sr:Ca ratios across vertebrae sections were compared to bottom salinity for fish collected near monitoring stations in the Caloosahatchee and Turner Rivers in south Florida (locations C and E in Figure 1). Daily salinity data were obtained from the South Florida Water Management District's Cape Coral Bridge station in the Caloosahatchee River and from the USGS's Turner River hydrographic station in Everglades National Park (ENP). Sr:Ca data from vertebrae sections had to be converted from distance across the vertebrae to estimated date. This was accomplished by assuming a 15 April birth date, 1 January as the date when opaque bands began forming (see Results), and constant growth between opaque bands. A birth date of 15 April was assumed given the peak appearance of neonate sawfish in south Florida gillnet sampling conducted to estimate juvenile abundance [21], [22].

### Growth estimation

The first opaque band on each vertebra section was assumed to be the natal mark [23], [12] and thereafter opaque bands were assumed to be formed annually (see Results). Under those assumptions, integer age equals n-1 opaque zones. Fractional age was estimated for each fish based on the assumptions of mean birth date being 15 April [22], and opaque band formation beginning 1 January. Estimated total days alive then were divided by 365 to estimate the fractional age of each sampled fish. A von Bertalanffy growth function (VBGF) was fit to size at fractional age data with the method of least squares computed with Proc NLIN in SAS [24], [25]:
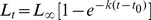
(1)where: L_t_  =  total length L_∞_  =  the length asymptote, k =  Brody's growth coefficient, t =  age in years, and t_0_  =  hypothetical age at which length is zero.

## Results

**Figure 2:**
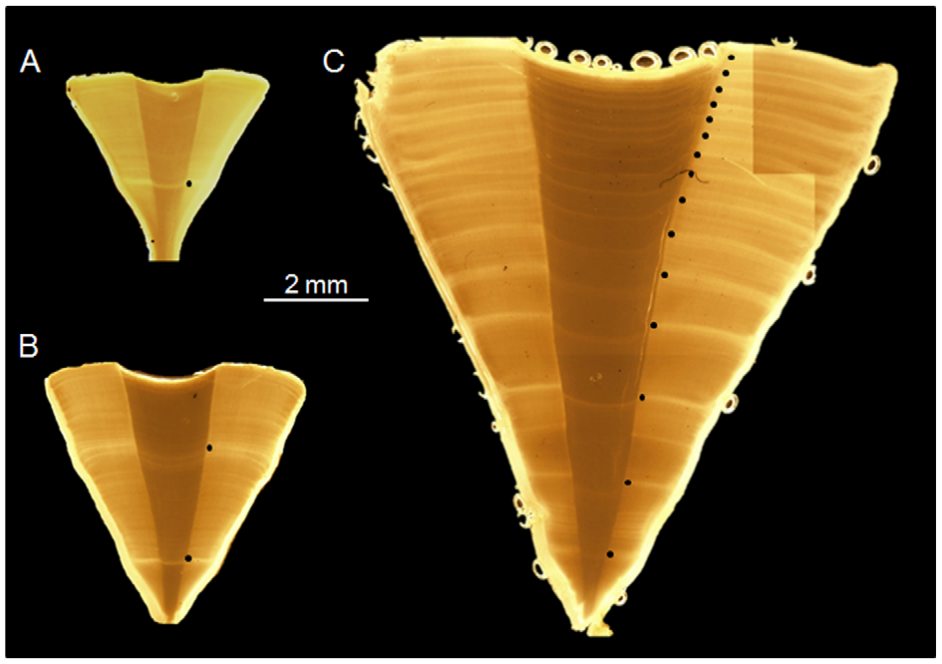
Thin sections of vertebrae from three sawfish. Digital images of thin sections of smalltooth sawfish vertebrae centra from carcasses opportunistically sampled in south Florida from 2003 through 2012. Vertebrae were from A) a 1.50 m TL male (fish 8 in Table 1), B) a 2.22 m TL female (fish 7), and C) a 4.35 m TL female (fish 13). Black circles indicate opaque zones, with the first opaque zone in each section being the natal mark following birth.

Vertebrae from 15 smalltooth sawfish collected in south Florida between 2003 and 2012 were made available for use in this study (Table 1, Figure 1). Sawfish size ranged from a 0.60 m TL male to a 4.35 m TL female. Opaque bands were apparent and easily discernible in all vertebrae sections (Figure 2). Opaque band counts differed between readers by one band for four fish, resulting in an APE of 3.93%. All sawfish for which opaque margins were overlooked by one reader had been recovered in winter. Following re-examination of these sections, opaque band counts were assigned by consensus.

**Table 1:** Collection and biological information for 15 sawfish carcasses opportunistically sampled in south Florida from 2003 to 2012.

Sample	Date Collected(M/DD/YY)	Sex	Length (m)	Maturity	Location	Opaque Band Count	Estimated Age (years)
1	4/4/03	F	2.45	I	Charlotte Harbor (A)	3	2.0
2	7/24/07	M	3.08	I	Marquesas Islands (F)	6	5.3
3	8/30/07	M	0.60	I	Long Key (G)	1	0.4
4	5/28/08	F	1.70	I	Ten Thousand Islands (D)	2	1.1
5	4/20/09	F	4.33	M	Key Largo (H)	10	9.0
6	6/17/09	F	1.88	I	Caloosahatchee River (B)	2	1.2
7	1/19/10	F	2.22	I	Chokoloskee Bay (E)	3	1.8
8	1/27/10	M	1.50	I	Caloosahatchee River (C)	2	0.8
9	2/8/10	F	1.96	I	Caloosahatchee River (C)	3	1.8
10	2/10/10	F	1.96	I	Caloosahatchee River (C)	3	1.8
11	2/26/10	M	1.97	I	Caloosahatchee River (C)	4	2.9
12	8/19/10	M	1.32	I	Caloosahatchee River (B)	2	1.3
13	4/28/11	F	4.35	M	Hobe Sound (J)	15	14.0
14	6/4/11	F	4.15	M	St. Lucie Inlet (K)	11	10.1
15	1/12/12	M	3.81	M	Biscayne Bay (I)	11	10.8

Maturity: M =  mature, I =  immature. Exact locations of collection are provided on Figure 1.

### Age verification

**Figure 3:**
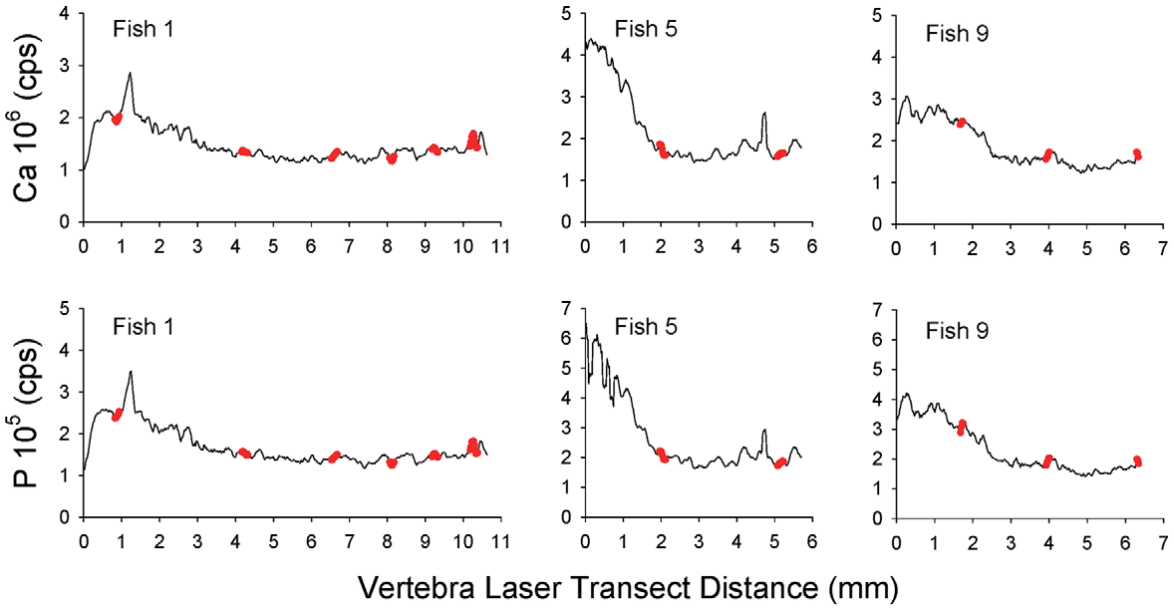
Trends in calcium versus phosphorus count data. Trends in Ca and P count data (cps  =  counts per second) from laser ablation-inductively coupled plasma-mass spectrometry transects across vertebral sections of three smalltooth sawfish. Data were smoothed by computing 5-spot moving averages prior to plotting lines. Red circles indicate location of opaque bands. Panel labels correspond to fish sample numbers in Table 1.

**Figure 4:**
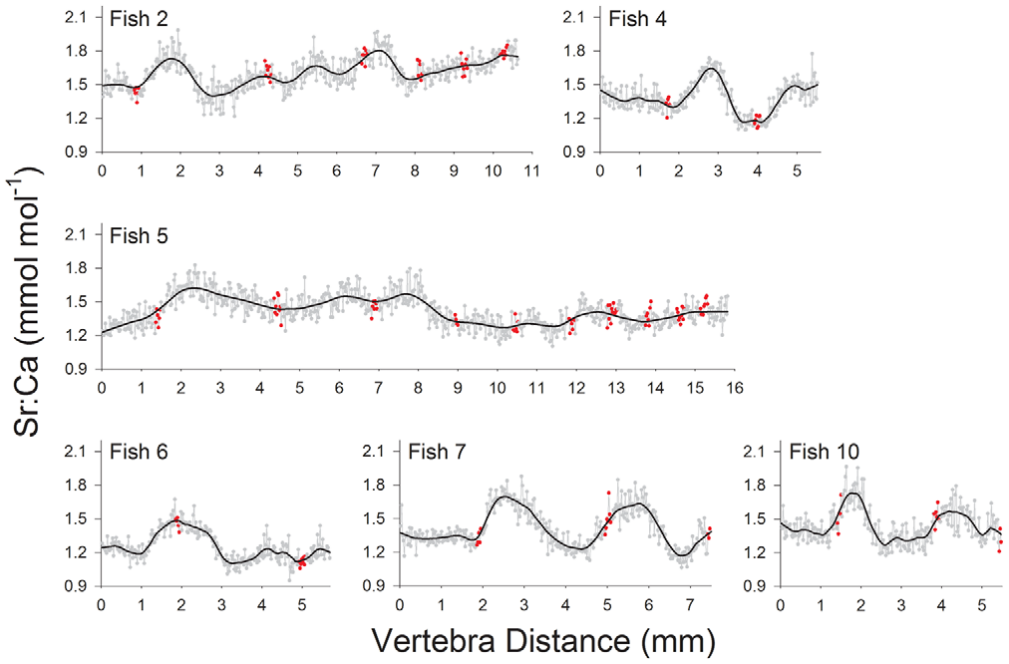
Trends in Sr:Ca across vertebrae sections. Trends in Sr:Ca across smalltooth sawfish vertebrae sections analyzed with laser ablation-inductively coupled plasma-mass spectrometry. Gray circles in each panel indicate individual measurements, while black lines indicate loess regression fits to the data. Red circles indicate location of subsequent opaque bands. Panel labels correspond to fish sample numbers in Table 1.

**Figure 5:**
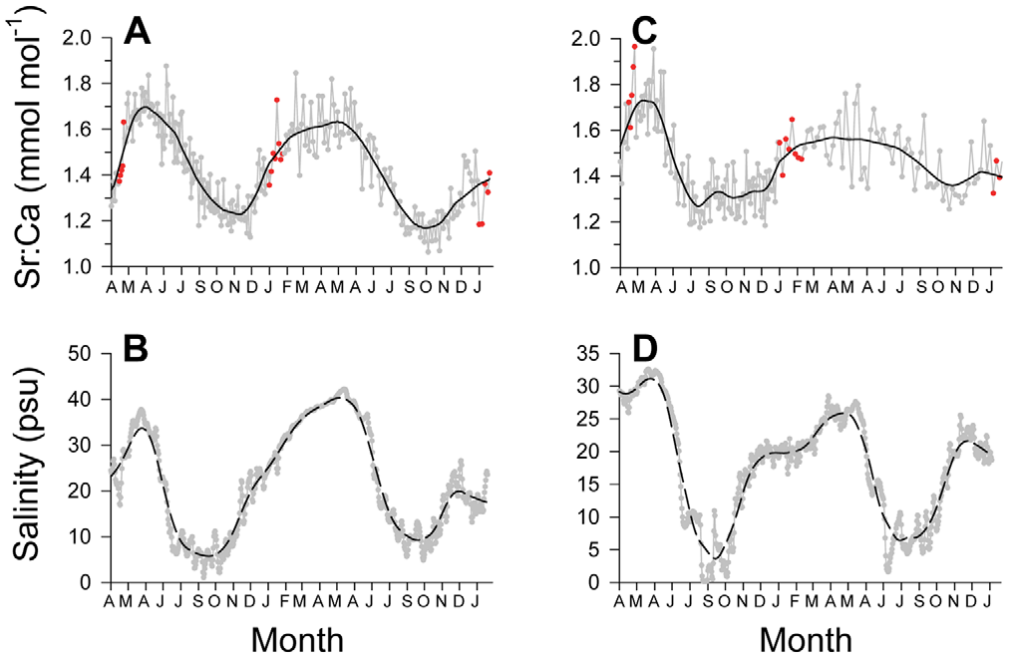
Association between Sr:Ca and estuarine nursery bottom salinity. Correspondence between Sr:Ca versus estimated date and bottom salinity versus date A) a 1.96 m female smalltooth sawfish (fish 10 in Table 1) whose carcass was collected in the Caloosahatchee River. B) Continuous bottom salinity data in the Caloosahatchee River during the time period depicted in A. C) a 2.22 m female (fish 7) whose carcass was collected near the mouth of the Turner River in Chokoloskee Bay. D) Continuous bottom salinity data in the Turner River during the time period depicted in C. Sr:Ca data are presented from the natal mark to the edge of the vertebra section. Gray circles in each panel indicate individual measurements, red circles indicate opaque bands, and black lines indicate loess regression fits to the data.

Calcium and P counts from LA-ICP-MS analysis of sawfish vertebrae were highly correlated (Pearson's correlation; *r* = 0.99; *p*<0.001). However, no relationship was apparent between peaks in Ca or P counts and opaque bands (Figure 3). This resulted from Ca or P not being highly variable between adjacent opaque and translucent bands within vertebrae. A cyclical pattern of Sr:Ca ratios was apparent between opaque bands among all vertebrae, but the range in ratios tended to be greater earlier rather than later in life (Figure 4). There was a high correspondence between Sr:Ca signatures and river (nursery) salinity for sawfish recovered near water monitoring stations in the Caloosahatchee and Turner Rivers (Figure 5). Therefore, Sr:Ca ratios in sawfish vertebrae appear to have recorded the seasonal trends observed in river salinity between adjacent opaque bands, a pattern that suggests annual formation of opaque bands.

### Growth estimation

**Figure 6:**
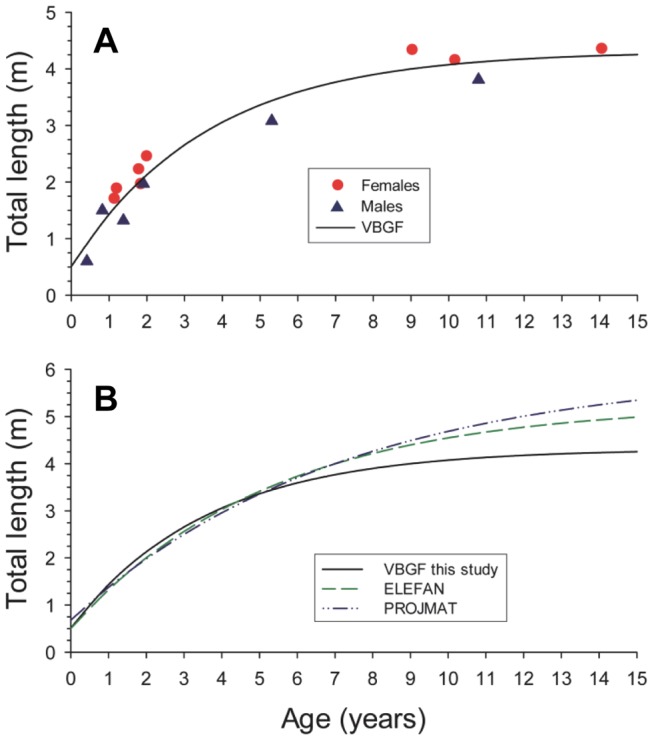
Von Bertalanffy growth functions for smalltooth sawfish. A) Size at fractional age for smalltooth sawfish samples collected in south Florida and aged via analysis of vertebrae centra thin sections. Plotted line is a von Bertalanffy growth function (VBGF) fit to the data with the method of least squares. Parameter estimates for the function are 4.48 m for L_∞_, 0.219 y^−1^ for k, and −0.81 years for t_0_. B) Comparison of VBGF function estimated in this study with those estimated by Simpfendorfer et al. [11] with length frequency data (ELEFAN seasonal; PROJMAT non-seasonal).

Fractional age estimates ranged from 0.4 to 14.0 years (Table 1). Ten of 15 samples were less than 3 years old. Ages, for the four largest fish (3.81–4.35 m TL), were between 9.0 y and 14.0 y. The VBGF computed with the method of least squares was statistically significant (non-linear regression; *R* ^2^  = 0.94; *p*<0.001). Parameter estimates (±95% confidence intervals) for the function are 4.48 m (±0.80 m) for L_∞_, 0.219 y^−1^ (±0.153 y^−1^) for k, and −0.81 y (±1.12 y) for t_0_ ( ). Predicted size at age from growth functions reported by Simpfendorfer et al. [11], correspond well to the VBGF computed with vertebrae-derived ages for fish <5 years old, but growth estimates diverge thereafter (Figure 6B).

## Discussion

Opaque bands were clear and easy to discern in smalltooth sawfish vertebrae sections. As sawfish got older, outer bands became more tightly spaced, which is a common phenomenon when aging elasmobranchs [12], but were still distinguishable. Given the likelihood that maximum age of smalltooth sawfish could be decades [26], it is unknown if band deposition stops in older sawfish when somatic growth ceases as was reported for porbeagle (*Lamna nasus*) [27] and sandbar sharks (*Carcharhinus plumbeus*) [28]. Nonetheless, our initial observations indicated that vertebrae centra may serve as useful aging structures for sawfish. However, validation or verification of annual formation of opaque zones is required to infer age from fish hard parts such as elasmobranch vertebrae [16]–[18].

Traditional age verification techniques, such as marginal increment analysis, could not be conducted in this study due to small sample size. However, marginal condition of smalltooth sawfish vertebrae sections was consistent with a single opaque band being formed each year. Vertebrae of fish collected in winter had opaque bands on the edge of their vertebrae sections, indicating opaque bands were being formed at the time these fish died. Vertebrae of fish recovered during other seasons did not have opaque edges, thus were clearly in the middle of translucent band growth.

An attempt was made to apply the approach of Hale et al. [19] for more robust age verification. Unfortunately, there was no correspondence between position of opaque bands and peaks in Ca and P count data from LA-ICP-MS analysis. Trends observed in Sr:Ca across vertebrae sections, however, mirrored the wet and dry seasons which drive seasonal salinity trends in south Florida [29]–[31] where the US sawfish population is currently concentrated [5]–[8]. We infer that this correspondence links vertebral Sr:Ca to ambient salinity, a relationship which has been clearly demonstrated for bony fish otoliths, scales, and fin rays [32]–[34]. Furthermore, we propose that the intra-annual trend in estuarine nursery salinity serves as a chronometer to verify annual formation of opaque zones in vertebrae given the correspondence between salinity and vertebral Sr:Ca trends.

Trends observed in Sr:Ca across vertebrae suggest natural tags formed in smalltooth sawfish vertebra centra reflect salinity experienced by the fish, a phenomenon that has also been reported recently for euryhaline bull (*Carcharhinus leucas*) and pigeye (*Carcharhinus amboinensis*) sharks in Australia [35], [36]. Sr:Ca ratios in sawfish vertebrae oscillated widely until age 2–2.5 y, after which lower variability was observed. This pattern suggests young juveniles experience a wider range of salinity than older juveniles and adults, which is supported by direct observations from the field [21], [22], [37], [38]. In recent sampling in south Florida, juvenile (<3 y) sawfish were recorded in salinities from 0 to 40 psu, while adults are typically observed in open-water habitats with more stable oceanic salinities (∼35 psu) such as outer Florida Bay or off the Atlantic side of the Florida Keys [8],[21],[22]. Furthermore, results of acoustic telemetry studies in the Caloosahatchee River, Florida indicate smaller (<1.5 m TL) acoustically-tagged juvenile smalltooth sawfish displayed limited movement thus were exposed to maximum seasonal fluctuations in salinity, while larger juveniles (>1.5 m TL) moved greater distances [37], [38]. Not only do telemetry data match the observations of Bethea et al. [21] from their field sampling, they are also consistent with the greater variability reported here in sawfish vertebral Sr:Ca in early life if salinity is the key factor driving Sr:Ca incorporation in vertebrae. Collectively, our Sr:Ca data and results from earlier telemetry and nursery habitat studies suggest that smalltooth sawfish leave their estuarine nurseries and move to higher salinity coastal waters by the end of their third year.

Research on Sr or other trace metal incorporation into elasmobranch vertebrae is relatively new [35], [36], but has been well-studied for bony fish otoliths [39], [32]. However, the difference in matrices between otoliths and vertebrae make these two hard parts difficult to compare. Elasmobranch vertebrae have a highly calcified hydroxyapatite matrix, while otoliths are acellular structures composed principally of biogenic calcium carbonate (aragonite). The matrix of vertebrae centra is similar to that of mammalian bone, in which Sr also has been shown to replace Ca [40]. Furthermore, recent research on the element chemistry of biogenic apatite structures in bony fish, such as scales and fin rays, indicates that apatite, composed primarily of calcium phosphate, effectively incorporates divalent cations (e.g., Sr, Ba, Mg, Mn) from water by substitution for Ca, similar to substitution by these cations for Ca in the aragonite matrix of otoliths [33], [34]. Clearly, controlled experiments need to be conducted with other, non-endangered elasmobranchs to test the effect of various factors on the incorporation of trace metals in vertebrae. However, the present lack of such studies does not preclude the inference here that salinity and vertebrae Sr:Ca are linked, and that the intra-annual cycle of salinity in south Florida sawfish nurseries serves as a chronometer to verify the annual formation of opaque bands in sawfish vertebrae.

Based on the conclusion that opaque zones are formed annually in smalltooth sawfish vertebrae, the oldest sawfish observed in this study was 14.0 y for a 4.35 m TL female. It should be noted, however, that fish have been observed in the wild approaching 6 m [7], thus it is unclear what the maximum longevity is for this species. Simpfendorfer [7] estimated that smalltooth sawfish may be capable of living several decades. While the oldest smalltooth sawfish aged in the current study was just a teenager, Tanaka [23] aged the congener *P. microdon* to 42 y. Given that observation, the fact that the largest sawfish in our sample was approximately 60% of maximum size, and the shape of the VBGF estimated here for smalltooth sawfish, it is likely this species can live longer than 14 y as well.

Von Bertalanffy growth parameters reported here indicate smalltooth sawfish may grow faster than previously estimated. Simpfendorfer et al. [11] modeled smalltooth sawfish growth from tag-recapture and length frequency data. Their best model fits produced VBGF parameters of 6.00 m for L_∞_, 0.140 y^−1^ for k, and −0.86 y for t_0_ (PROJMAT non-seasonal model) from length frequency data, and 5.27 m for L_∞_, 0.189 y^−1^ for k, and −0.53 y for t_0_ (ELEFAN seasonal model) from length frequency data. They also modeled juvenile sawfish growth with tag-recapture data, but were unable to estimate VBGF parameters because no recapture data existed for fish greater than 2.2 m TL. The VBGFs they did produce predict similar size at age for young fish (<5 y) as the VBGF computed here, which serves as another form of age verification for opaque band counts in vertebrae. However, key differences among functions are a lower L_∞_ and higher k estimated from size at age data reported here. It is possible that these differences are due to the fact that mostly juvenile data were modeled by Simpfendorfer et al. [11], thus L_∞_ was overestimated, which in turn led to a more moderate slope and lower k for their function. Alternatively, the limited sample size in the current study may have biased results reported here if size at age data do not reflect the population as a whole. Uncertainty exists in model parameters due to wide confidence limits resulting from small sample size. However, the high coefficient of determination (*R* ^2^ = 0.94) indicates the VBGF fits smalltooth sawfish size at age data well. Furthermore, the model predicts smalltooth sawfish size at age-0 to be 0.73 m, which is the midpoint of the range of size at birth (0.67–0.81 m) reported by Poulakis et al. [22] based on neonatal smalltooth sawfish that still had partial rostral sheaths present.

This study has added to our understanding of smalltooth sawfish life history and ecology, as well as introduced new techniques to aid in its conservation and recovery. While growth functions had been estimated previously for smalltooth sawfish, direct age estimates have not been available until now. This allowed the estimation of VBGF parameters directly from size at age data, which is the preferred approach. Previous estimates of population recovery rates lacked information about life history parameters [26], [41]. Direct estimates of smalltooth sawfish growth now can be incorporated into productivity models to estimate the intrinsic rate of population increase and project population recovery.

Lastly, LA-ICP-MS results reported here have important implications for examining habitat utilization in other elasmobranchs that have nurseries in estuarine or freshwater habitats, as well as for tracking movement patterns and salinity history for euryhaline adult elasmobranchs. However, controlled experiments must be conducted to test factors that may affect the incorporation of Sr and other trace elements into elasmobranch vertebrae.

## Acknowledgments

Samples were obtained under ESA permits #1352 (Mote Marine Laboratory), #1475 (Florida Fish and Wildlife Conservation Commission), and #13330 (NMFS Southeast Fisheries Science Center). Special thanks go to Simon Gulak, George Burgess, Beau Yeiser, Amy Timmers, Corey Keller, Lisa Hallock, Jackie DeAngelo, Sarah Erickson, and Jason Seitz for collecting, archiving, and making available sawfish vertebrae. Tracy Ziegler (Everglades National Park), Officers Randy Irwin, Jim Fillip, and Marc Shea (FWC Law Enforcement), Officer Robert E. Lee (St. Lucie County Sheriff), Michael Hankins (Loggerhead Club & Marina, Miami), and Richard Ray (Tarpon Point Marina, Cape Coral) coordinated recovery of carcasses so they could be studied. We thank Mike Colucci for providing access to the laser ablation system and Michael Cochran for technical assistance with LA-ICP-MS analysis.

## References

[1] Beissinger SR, McCullough R (2002) Population Viability Analysis. Chicago: University of Chicago Press.

[2] Cortés E (2002) Incorporating uncertainty into demographic modeling: application to shark populations and their conservation. Conserv Biol 16: 1048–1062.

[3] International Union for Conservation of Nature (IUCN) (2012) IUCN Red List of Threatened Species. Version 2010.1. Available: www.iucnredlist.org. Accessed 2012 March 11.

[4] Bigelow HB, Schroeder WC (1953) Fishes of the Western North Atlantic. Sawfishes, Guitarfishes, Skates, Rays, and Chimaeroids. Mem Sears Found Mar Res 1, part 2. New Haven: Yale University Press. 514 p.

[5] Seitz JC, Poulakis GR (2002) Recent occurrence of sawfishes (Elasmobranchiomorphi: Pristidae) along the southwest coast of Florida (USA). Florida Scient 65: 256–266.

[6] Seitz JC, Poulakis GR (2006) Anthropogenic effects on the smalltooth sawfish (*Pristis pectinata*) in the United States. Mar Pollut Bull 52: 1533–1540. 17034820 10.1016/j.marpolbul.2006.07.016

[7] Simpfendorfer CA (2002) Smalltooth sawfish: the USA's first endangered elasmobranch? Endang Spec Update 19: 45–49.

[8] Poulakis GR, Seitz JC (2004) Recent occurrence of the smalltooth sawfish, *Pristis pectinata* (Elasmobranchiomorphi: Pristidae), in Florida Bay and the Florida Keys, with comments on sawfish ecology. Florida Scient 67: 27–35.

[9] National Marine Fisheries Service (NMFS) (2003) Endangered and threatened species; final endangered status of a distinct population segment of smalltooth sawfish (*Pristis pectinata)* in the United States. US Federal Register 68: 15674–15680.

[10] Norton SL, Wiley TR, Carlson JK, Frick AL, Poulakis GR, et al (2012) Designating critical habitat for juvenile endangered smalltooth sawfish in the United States. Mar Coast Fish 4: 473–480.

[11] Simpfendorfer CA, Poulakis GR, O'Donnell PM, Wiley TR (2008) Growth rates of juvenile smalltooth sawfish *Pristis pectinata* Latham in the western Atlantic. J Fish Biol 72: 711–723.

[12] Cailliet GM, Goldman KJ (2004) Age determination and validation in. chondricthyan fishes. In: Carrier J, Musick JA, Heithaus MR, editors. Biology of sharks and their relatives. Boca Raton: CRC Press, 399–447.

[13] Chen CT, Leu TC, Joung SJ, Lo NCH (1990) Age and growth of the scalloped hammerhead, *Sphyrna lewini,* in northeastern Taiwan waters. Pac Sci 44: 156–170.

[14] Natanson LJ, Wintner SP, Johansson F, Piercy A, Campbell P, et al (2008) Ontogenetic vertebral growth patterns in the basking shark, *Cetorhinus maximus.* . Mar Ecol Prog Ser 361: 267–278.

[15] Baremore IE, Andrews KI, Hale LF (2009) Difficulties associated with modeling growth in the Atlantic angel shark (*Squatina dumeril*). Fish Res 99: 203–209.

[16] Beamish RJ, McFarlane GA (1983) The forgotten requirement for age validation in fisheries biology. Trans Am Fish Soc 112: 735–743.

[17] Campana SE (2001) Accuracy, precision and quality control in age determination, including a review of the use and abuse of age validation methods. J Fish Biol 59: 197–242.

[18] Cailliet GM, Smith WD, Mollet HF, Goldman KJ (2006) Age and growth studies. of chondricthyan fishes: the need for consistency in terminology, verification, validation and growth function fitting. Environ Biol Fish 77: 211–228.

[19] Hale LF, Dudgeon JV, Mason AZ, Lowe CG (2006) Elemental signatures in the. vertebral cartilage of the round stingray, *Urobatis halleri,* from Seal Beach, California. Environ Biol Fish 77: 317–325.

[20] Beamish RJ, Fournier DA (1981) A method for comparing the precision of a set of age determinations. Can J Fish Aquat Sci 38: 982–983.

[21] Bethea DM, LaPorte A, Carlson JK (2009) NOAA NMFS smalltooth sawfish monitoring report-FY-09: Relative abundance and essential fish habitat studies for smalltooth sawfish, *Pristis pectinata,* in Southwest Florida, USA. Southeast Fisheries Science Center Panama City Laboratory.

[22] Poulakis GR, Stevens PW, Timmers AA, Wiley TR, Simpfendorfer CA (2011) Abiotic affinities and spatiotemporal distribution of the endangered smalltooth sawfish, *Pristis pectinata,* in a south-western Florida nursery. Mar Freshw Res 62: 1165–1177.

[23] Tanaka S (1991) Age estimation of freshwater sawfish and sharks in Northern Australia and Papua New Guinea. Univ Mus, Univ Tokyo, Nat Cult 3: 71–82.

[24] von Bertalanffy L (1938) A quantitative theory of organic growth (inquiries on growth laws II). Hum Biol 10: 181–213.

[25] SAS Institute Inc (2004) SAS/STAT 9.1 User's Guide. Cary, NC: SAS Institute Inc.

[26] Simpfendorfer CA (2000) Predicting recovery rates for endangered western Atlantic sawfishes using demographic analysis. Environ Biol Fish 58: 371–377.

[27] Francis MP, Campana SE, Jones CM (2007) Age under-estimation in New Zealand 29. porbeagle sharks (*Lamna nasus*): is there an upper limit to ages that can be determined from shark vertebrae? Mar Freshw Res 58: 10–23.

[28] Andrews AH, Natanson J, Kerr LA, Burgess GH, Cailliet GM (2011) Bomb radiocarbon and tag-recapture dating of sandbar shark (*Carcharhinus plumbeus*). Fish Bull 109: 454–465.

[29] Dolan TJ, Hermann AJ, Bayley SE, Zoltek Jr J (1984) Evapotranspiration of a Florida, USA, freshwater wetland. J Hydro 74: 355–371.

[30] Faunce CH, Serafy JE, Lorenz JL (2004) Density-habitat relationships of mangrove creek fishes within the southeastern saline Everglades (USA), with reference to the managed freshwater releases. Wetl Ecol Manag 12: 377–394.

[31] Richardson CJ (2010) The Everglades: North America's subtropical wetland. Wetl Ecol Manag 18: 517–542.

[32] Secor DH, Rooker JR (2000) Is otolith strontium a useful scalar of life cycles in. estuarine fishes? Fish Res 46: 359–371.

[33] Wells BK, Rieman BE, Clayton JL, Horan DL, Jones CM (2003) Relationships between water, otolith, and scale chemistries of westslope cutthroat trout from the Coeur d'Alene River, Idaho: the potential application of hard-part chemistry to describe movements in freshwater. Trans Am Fish Soc 132: 409–424.

[34] Phelps QE, Whitledge GW, Tripp SJ, Smith KT, Garvey JE (2012) Identifying river of origin for age-0 *Scaphirhynchus* sturgeons in the Missouri and Mississippi rivers using fin ray microchemistry. Can J Fish Aquat Sci 69: 930–941.

[35] Tillett BJ, Meekan MG, Parry D, Munksgaard N, Field IC, et al (2011) Decoding fingerprints: elemental composition of vertebrae correlates to age-related habitat use in two morphologically similar sharks. Mar Ecol Prog Ser 434: 133–142.

[36] Werry JM, Lee SY, Otway NM, Hu Y, Sumpton W (2011) A multifaceted approach for quantifying the estuarine nearshore transition in the life cycle of the bull shark, *Carcharhinus leucas.* . Mar Freshw Res 62: 1421–1431.

[37] Simpfendorfer CA, Yieser BG, Poulakis GR, Stevens PW, Heupel MR (2011) Environmental influences on the spatial ecology of juvenile smalltooth sawfish (*Pristis pectinata*): results from acoustic monitoring. PLoS One 6: e16918. 21347294 10.1371/journal.pone.0016918 PMC3037940

[38] Poulakis GR, Stevens PW, Timmers AA, Stafford CJ, Simpfendorfer CA (2012) Movements of juvenile endangered smalltooth sawfish, *Pristis pectinata,* in an estuarine river system: use of non-main-stem river habitats and lagged responses to freshwater inflow-related changes. Environ Biol Fish.

[39] Campana SE (1999) Chemistry and composition of fish otoliths: pathways, mechanisms and applications. Mar Ecol Prog Ser 188: 263–297.

[40] Boivin G, Deloffre P, Perrat B, Panczer G, Boudeulle M, et al (1996) Strontium distribution and interactions with bone mineral in monkey iliac bone after strontium salt (S 12911) administration. J Bone Miner Res 11: 1302–1311. 8864905 10.1002/jbmr.5650110915

[41] Carlson JK, Osborne TW, Schmidt TW (2007) Monitoring the recovery of smalltooth sawfish, *Pristis pectinata,* using standardized relative indices of abundance. Biol Conserv 136: 195–202.

